# Effect of Mailing an At-home Disposal Kit on Unused Opioid Disposal After Surgery: A Randomized Clinical Trial

**DOI:** 10.1001/jamanetworkopen.2022.10724

**Published:** 2022-05-06

**Authors:** Anish K. Agarwal, Daniel Lee, Zarina Ali, Yaxin Wu, Mary Cognilio, Tanya Uritsky, M. Kit Delgado

**Affiliations:** 1 Department of Emergency Medicine, University of Pennsylvania, Philadelphia; 2 Leonard Davis Institute of Health Economics, University of Pennsylvania, Philadelphia; 3 Penn Medicine Center for Health Care Innovation, University of Pennsylvania, Philadelphia; 4 Division of Urology, Department of Surgery, University of Pennsylvania, Philadelphia; 5 Department of Neurosurgery, University of Pennsylvania, Philadelphia; 6 Penn Medicine, University of Pennsylvania Health System, Philadelphia; 7 Department of Biostatistics, Epidemiology, and Informatics, University of Pennsylvania, Philadelphia

## Abstract

This randomized clinical trial tests the effect of a mailed, at-home disposal kit on disposal rates of unused opioids after surgery.

## Introduction

Many prescription opioids after surgery are left unused and are at risk of being misused.^1,2^ Encouraging proper disposal is important, yet motivating this behavior remains challenging because patients must understand the risks of opioid use and the benefits of disposal and identify methods of safe disposal.^3^ We sought to test the effect of a mailed at-home disposal kit on disposal rates of unused opioids after surgery.

## Methods

This 2-arm, randomized clinical trial compared usual care vs a mailed at-home disposal kit that was embedded in an existing postoperative text messaging program designed to capture patient-reported outcomes data and self-reported disposal.^4^ The trial protocol is available in Supplement 1. The study was approved by the University of Pennsylvania Institutional Review Board with a waiver of informed consent. The trial took place from April 19 to June 1, 2021, and followed the CONSORT reporting guideline.

All patients 18 years or older undergoing an orthopedic or urologic procedure and prescribed an opioid replied through a text messaging platform using an established protocol.^5^ Patients without access to a short messaging service–capable device or who did not speak English were excluded. Participants were electronically block randomized in groups of 2 or 4 to usual care or a mailed at-home disposal kit immediately after consent (eMethods in Supplement 2). Usual care consisted of a text message hyperlink to nearby disposal locations. Intervention participants were mailed an at-home disposal kit timed to arrive on postoperative day 4 to 7 based on prior data and designed to provide patients with a disposal method when they were most likely to be finished taking opioids.^5^ The disposal kit (DisposeRx Inc.) sequesters tablets in a polymer gel. Patients self-reported disposal via text messaging. Baseline self-reported disposal rates were 25% to 30%. We estimated 75 patients per group, assuming a 2-sided α = .025, a β of 80%, and a 50% relative increase in self-reported disposal. The primary outcome of interest was self-reported disposal. All randomized participants were included in an intention-to-treat analysis, and statistical analyses were performed using Fisher exact and *χ*^2^ tests via R, version 3.60 (R Project for Statistical Computing).

## Results

Of 657 patients invited to participate, 302 (46.0%) consented. Demographic information was obtained from the electronic health record to evaluate characteristics of respondents and nonrespondents. Participants had a median age of 56.5 (IQR, 40.0-65.8) years; 136 (45.0%) were women and 166 (55.0%) were men; 240 (79.5%) were White and 44 (14.6%) were Black; and 235 (77.8%) completed all questions. Consenting participants were younger than individuals who declined or did not respond (median age, 59.0 [IQR, 47.0-68.0] years), and more consenting participants than nonparticipants were White (215 of 355 [60.6%]). Participants were more likely than nonparticipants to be opioid naive (225 of 302 [74.5%] vs 225 of 355 [63.4%]) (Table 1). The odds of self-reported disposal were 2.01 (95% CI, 1.2-3.4) times higher in the intervention group. Among the 235 participants who completed all questions, 47 of 110 (42.7%) in the usual care group self-reported disposal, compared with 75 of 125 (60.0%) in the intervention group (Table 2). An estimated additional 480 unused opioid tablets were disposed of in the intervention group.

**Table 1.** Participant Characteristics^a^

Characteristic	Participant group		No response or declined (n = 355)	*P* value
	Intervention (n= 159)	Control (n = 143)		
Age, median (IQR), y	55.0 (36.5-65.5)	57.0 (41.0-65.5)	59.0 (47.0-68.0)	.01
Sex				
Women	71 (44.7)	65 (45.5)	164 (46.2)	.76
Men	88 (55.3)	78 (54.5)	189 (53.2)	
Race				
American Indian	1 (0.6)	0	0	.002
Asian	4 (2.5)	2 (1.4)	12 (3.4)	
Black	26 (16.4)	18 (12.6)	108 (30.4)	
East Indian	0	0	2 (0.6)	
White	124 (78.0)	116 (81.1)	215 (60.6)	
Other^b^	3 (1.9)	4 (2.8)	8 (2.3)	
Patient declined	0	1 (0.7)	3 (0.8)	
Unknown	1 (0.6)	2 (1.4)	5 (1.4)	
Hispanic ethnicity				
Black	1 (0.6)	0	4 (1.1)	.09
White	1 (0.6)	0	10 (2.8)	
Anxiety	15 (9.4)	10 (7.0)	38 (10.7)	.50
Opioid naive	117 (73.6)	108 (75.5)	225 (63.4)	.03
Opioid use disorder	0	1 (0.7)	5 (1.4)	.38
Alcohol use disorder	2 (1.3)	0	4 (1.1)	.49
Smoker	4 (2.5)	2 (1.4)	9 (2.5)	.71
NSAID use	52 (32.7)	61 (42.7)	126 (35.5)	.27

Abbreviation: NSAID, nonsteroidal anti-inflammatory drug.

^a^ Unless otherwise indicated, data are expressed as number (%) of participants.

^b^ Includes Native Hawaiian, other Pacific Islander, and more than 1 race.

**Table 2.** Self-reported Opioid Disposal

Characteristic	Participant group^a^	
	Usual care (n = 110)	Intervention (n = 125)
Self-reported disposal	47 (42.7)	75 (60.0)
Day		
4	16 (14.5)	17 (13.6)
7	18 (16.4)	26 (20.8)
14	7 (6.4)	27 (21.6)
21	6 (5.5)	5 (4.0)
28	0	0
Day of disposal, median (IQR)	7 (4-14)	7 (7-14)

^a^ Includes the 235 participants who completed all questions. Unless otherwise indicated, data are expressed as number (%) of participants.

## Discussion

Opioid disposal remains important, but motivating disposal remains challenging. In this randomized clinical trial, a mailed at-home disposal kit increased self-reported opioid disposal rates after common surgical procedures. Efforts to encourage disposal are growing, yet research in scalable and patient-centered approaches is lacking. The demonstrated impact has high potential for a low cost solution. This study is limited in that it relied on self-reported disposal and reflects a single center’s experience; in addition, consenting patients were more likely to be younger and White, which limits generalizability. However, the findings build on literature suggesting that providing patients with at-home disposal options can improve self-reported disposal rates.^6^ The process of mailing the disposal kit is simple, inexpensive (approximately $1.50 per mailed kit), and scalable.

## Notes

Supplement 1.
Trial Protocol

Supplement 2. **eMethods.** Study Population, Data Collection, Statistical Analysis, and Study Flowchart
eReferences

Supplement 3.
Data Sharing Statement
