# Error in Byline and Affiliations

**DOI:** 10.1001/jamanetworkopen.2023.19834

**Published:** 2023-06-06

In the Research Letter titled “Effect of Mailing an At-home Disposal Kit on Unused Opioid Disposal After Surgery: A Randomized Clinical Trial,”^1^ published May 6, 2022, Mary Coniglio’s surname was misspelled in the byline and author affiliations. This article has been corrected.
