# Supplementary material for: Effect of Mailing an At-home Disposal Kit on Unused Opioid Disposal After Surgery: A Randomized Clinical Trial
Source: JAMA Netw Open. 2022 May 6;5(5):e2210724. doi: 10.1001/jamanetworkopen.2022.10724 (PMC9077482; doi:10.1001/jamanetworkopen.2022.10724)
Supplement: Supplement 1. — Trial Protocol [file jamanetwopen-e2210724-s001.pdf]

Study Protocol

Table of Contents

1. Summary of changes to the protocol

2. Original Study Protocol

3. Final Study Protocol

Study Protocol

A Randomized, Controlled Trial of Mailed Opioid Disposal Kits to Improve Post-Operative Opioid Disposal

Study Protocol

March 18, 2021

Outline

1. Abstract

2. Overall objectives

3. Aims

    3.1 Primary outcome

    3.2 Secondary outcomes

4. Background

5. Study design

    5.1 Design

    5.2 Study duration

    5.3 Target population

    5.4 Accrual

    5.5 Key inclusion criteria

    5.6 Key exclusion criteria

6. Subject recruitment

7. Subject compensation

8. Study procedures

    8.1 Consent

    8.2 Procedures

9. Analysis plan

10. Investigators

11. Human research protection

    11.1 Data confidentiality

    11.2 Subject confidentiality

    11.3 Subject privacy

    11.4 Data disclosure

    11.5 Data safety and monitoring

11.6 Risk/benefit

11.6.1 Potential study risks

11.6.2 Potential study benefits

11.6.3 Risk/benefit assessment

1. Abstract

The majority of acute, short-term opioid tablets prescribed following surgery go unused. In a recent analysis of post-operative orthopedic and urologic surgery data at 7 days >60% of tablets were left unused, and patients reported no intention of using them, by post-operative day 7. Disposal of unused opioid medications is important to limit diversion, misuse, and conversion to new persistent use. These are salient to the patient and others around them including children, family and friends. Though patients can flush their unused opioids, early research has shown that providing patients with disposal techniques can improve proper disposal rates. In this proposal we seek to test, through a randomized clinical trial, if providing patients with an opioid disposal kit at key post-operative times can improve the rate of self-reported disposal as compared with usual care. Using an established, automated system our primary outcome will be self-reported opioid disposal rates. We will conduct a prospective RCT amongst patients undergoing urologic surgeries, neurosurgeries, and orthopedic surgeries.

2. Overall objectives

The objective of this study is to use a randomized, controlled trial to test the effectiveness of mailed post-operative opioid disposal to promote safe opioid disposal following procedures in neurosurgery, orthopedics, and urology.

3. Aims

3.1 Primary outcome

The primary outcome variable is rate of self-reported opioid disposal.

3.2 Secondary outcome

The secondary outcome is self-reported number of opioid tablets used following surgery.

4. Background

The majority of acute, short-term opioid tablets prescribed following surgery go unused. In a recent analysis of post-operative orthopedic and urologic surgery data at 7 days >60% of tablets were left unused, and patients reported no intention of using them, by post-operative day 7. Additionally, in a sample of neurosurgery patients, 64% of patients reported having excess tablets and not disposing of them follow surgery. Disposal of unused opioid medications is important to limit diversion, misuse, and conversion to new persistent use. These are salient to the patient and others around them including children, family and friends. Though patients can flush their unused opioids, early research has shown that providing patients with disposal techniques can improve proper disposal rates.

Human behavior, and the study of behavioral economics, may provide an opportunity to improve opioid disposal after surgery but remains understudied. The concept of availability bias, or a tendency to think or act

on things which are most present in one's mind, can be tested in this context. In this proposal we seek to test, through a randomized clinical trial, if providing patients with an opioid disposal kit at key post-operative times can improve the rate of self-reported disposal as compared with usual care. Using an established, automated system our primary outcome will be self-reported opioid disposal rates. We will conduct a prospective RCT amongst patients undergoing urologic surgeries, neurosurgeries, and orthopedic surgeries.

5. Study design

5.1 Design

This is a two-arm, randomized, controlled trial. The study will be conducted using an automated, remote text messaging platform (Way to Health) which delivers text messaging following orthopedic, neurosurgical, and urology procedures at the University of Pennsylvania.

The study team will identify potential participants from the electronic health record at the University of Pennsylvania Health System using Penn Data Store and Clarity, an EPIC reporting database. Patients undergoing a procedure in neurosurgery, orthopedics, or urology and who are prescribed an acute opioid will receive a text message four days following their discharge. This established system obtains remote, text-messaged based consent on post-operative day four. For those consenting, patients are queried on self-reported pain intensity, ability to manage pain, use of non-opioid analgesics, use of opioid analgesics, and opioid disposal. One a participant responds and consents on day four, the participant will be mailed an opioid disposal kit which includes an informational sheet and the chemical packet (DispoRx).

Participants will be randomly assigned to intervention or control according to calendar week of surgery in alternating weeks over a 6-week period. Participants randomized to the control arm will receive no other interventions.

5.2 Study duration

This is 6-week study.

5.3 Target population

Adult (18 years or older), English speaking patients, with access to a mobile (SMS capable device) undergoing orthopedic, urologic, or a neurosurgical procedure

5.4 Accrual

We estimate that a sample size of 250 participants (125 per arm) will provide at least 80% power using a conservative Bonferroni adjustment of the Type I error rate with a 2-sided α of 0.025 to detect a 20% change in disposal rates. Preliminary data suggest a baseline rate of 25%.

5.5 Key inclusion criteria

1) Adult (18 years or older), 2) English speaking patients, 3) access to a mobile (SMS capable device), 4) prescribed an acute opioid, 5) consenting to text message and 6) undergoing orthopedic, urologic, or a neurosurgical procedure

5.6 Key exclusion criteria

1) Conditions that would make participation infeasible such as inability to provide informed consent, illiteracy or inability to speak, read, and write English or 2) not providing virtual consent

6. Subject recruitment

The study team will identify potential participants from the electronic health record at the University of Pennsylvania Health System using Penn Data Store and Clarity, an EPIC reporting database.

7. Subject compensation

No participants will be compensated.

8. Study procedures

8.1 Consent

Upon recruitment, individuals will be sent a text message via the Way to Health platform with an opportunity to consent or decline. Participants have the ability to opt-out of text messaging and the study at any point by text messaging a response of "STOP." The current post-operative text messaging protocol has been approved by the QI IRB and has been in place with an average response rate of 50-60%. We will not change the questions or timing of messages being sent. The questions related to opioid disposal are pre-existing.

8.2 Procedures

After providing informed consent, participants will be sent messaging on post-operative days 4, 7, 14, 21, and 28. At each time point, patients are asked to self-report pain intensity (scale 0-10, 10 being severe), ability to manage pain (scale 0-10, 10 being very able to manage), and use of non-opioid and opioid analgesics. Patients who are using opioid analgesics are asked to report quantity used, projection of future use for acute pain. Patients reporting no further use are asked about disposal (Yes or No).

Patients will be mailed opioid disposal packets, DispoRx, which are small chemical polymers which individuals can pour directly into a pill bottle. The individual shakes the bottle and the medicaition is deactivated, rendered unusable and can be placed safely in the trash. We will mail these packets to individuals within the first 7 days for Orthopedic and Urology and timed for 21 days for Neurosurgery.

Participants will be randomly assigned at the individual level to receive a disposal packet. Participants randomized to the control arm will receive no other interventions.

9. Analysis plan

To compare sample characteristics between arms we will use t-tests or Wilcoxon rank-sum tests (F-tests or Kruskal-Wallis test) for continuous variables and Pearson chi square tests or Fisher's exact tests for categorical variables. In our primary analyses, we will compare rates of opioid disposal (baseline vs. 6-week study period). In the secondary analysis we will compare self-reported pain intensity, ability to manage pain, and opioid use. All hypothesis tests will be two-sided using a two-sided alpha of 0.025 as our threshold for statistical significance. We will use Stata and/or SAS to analyze the data.

10. Investigators

Anish K Agarwal, MD, MPH, MS is the Principal Investigator (PI) and is an Assistant Professor of Emergency Medicine at the Perelman School of Medicine. He has past experience leading randomized, clinical trials and observation trials using text messaging. He currently spends 65% of his effort on research and 35% on clinical and teaching activities.

11. Human research protection

11.1 Data confidentiality

The study will utilize a client-server deployed Data Management System (DMS) rather than a 'Store and Forward' database configuration, obviating research site database security concerns. Confidential participant information will be entered into the database. If this information exists on paper CRFs, it will be filed under lock and key, with generation of a participant ID. Thereafter, confidential information will be made available to authorized users only as specifically needed. No one can gain access to an individual MySQL database table unless explicitly granted a user ID, password, and specific access. Even those with user names and passwords cannot gain access to the tables that contain the identifying participant information.

No results will be reported in a personally identifiable manner. All tracking system data will be password-protected with several levels of protection. The first will allow access to the operating system of the computer. The second will allow access to the basic menus of the integrated system; within certain menu options, such as database browsing, a third password will be required. Our prior research employing similar precautions has demonstrated that these techniques are very successful in assuring the protection of subjects.

Each investigator and staff member involved in the proposed study will sign and adhere to a Standard Operating Procedure for managing participant data through the automated texting platform and has participated in required IRB/HIPAA compliance training. We will also continue to make use of password protection programs for all computerized records. In no instances will identifying information be publicly disclosed. Prior to conducting any analyses, all identifiers (e.g., names, medical record numbers, health plan enrollee numbers, birth dates, etc. will be removed. Results from this part of the investigation will be reported in aggregate. All participant identification will be removed from the data 3 years after the study has been completed.

11.2 Subject confidentiality

All participants will provide informed consent for access to these materials. The data to be collected include demographic data (e.g., age, sex, self-identified race), prescription data, self-reported pain, use and disposal. Research material that is obtained will be used for research purposes only. The same procedure used for the analysis of automated data sources to ensure protection of patient information will be used for the survey data, in that patient identifiers will be used only for linkage purposes or to contact patients. The study identification number, and not other identifying information, will be used on all data collection instruments. All study staff will be reminded to appreciate the confidential nature of the data collected and contained in these databases. The Penn Medicine Academic Computing Services (PMACS) will be the hub for the hardware and database infrastructure that will support the project and is where the Way to Health web portal is based. The PMACS is a joint effort of the University of Pennsylvania's Abramson Cancer Center, the Cardiovascular Institute, the Department of Pathology, and the Leonard Davis Institute. The PMACS provides a secure computing environment for a large volume of highly sensitive data, including clinical, genetic, socioeconomic, and financial information. Among the IT projects currently managed by PMACS are: (1) the capture and organization of complex, longitudinal clinical data via web and clinical applications portals from cancer patients enrolled in clinical trials; (2) the integration of genetic array databases and clinical data obtained from patients with cardiovascular disease; (3) computational biology and cytometry database management and analyses; (4) economic and health policy research using Medicare claims from over 40 million Medicare beneficiaries. PMACS requires all users of data or applications on PMACS servers to complete a PMACS-hosted cybersecurity awareness course annually, which stresses federal data security policies under data use agreements with the university. The curriculum includes Health Insurance Portability and Accountability Act (HIPAA) training and covers secure data transfer, passwords, computer security habits and knowledge of what constitutes misuse or inappropriate use of the server. We will implement multiple, redundant protective measures to guarantee the privacy and security of the participant data. All investigators and research staff with direct access to the identifiable data will be required to undergo annual responsible conduct of research, cybersecurity, and HIPAA certification in accordance with University of Pennsylvania regulations. All data for this project will be stored on the secure/firewalled servers of the PMACS Data Center, in data files that will be protected by multiple password layers. These data servers are maintained in a guarded facility behind several locked doors, with very limited physical access rights. They are also cyber-protected by extensive firewalls and multiple layers of communication encryption. Electronic access rights are carefully controlled by University of Pennsylvania system managers. We will use highly secure methods of data encryption for all transactions involving participants' financial information using a level of security comparable to what is used in commercial financial transactions. We believe this multi-layer system of data security, identical to the system protecting the University of Pennsylvania Health Systems medical records, greatly minimizes the risk of loss of privacy. In addition, risk of loss of confidentiality will be minimized by storing completed paper copies of the surveys and signed informed consent forms in locked file cabinets in locked offices accessible only to trained study staff. Each subject will be assigned a unique identifier without identifying information, and data will be entered into an electronic database using only the unique identifier. Only trained study staff will have access to the code that links the unique identifier to the subject's identity. Electronic data will be stored on secure, password-protected firewalled servers at the University of Pennsylvania.

11.3 Subject privacy

Interested participants will provide consent via text messaging. Enrollment will include a description of the voluntary nature of participation, the study procedures, risks and potential benefits in detail.. Participants will be

told that they do not have to answer any questions if they do not wish and can drop out of the study at any time, without affecting their medical care or the cost of their care. They will be told that they may or may not benefit directly from the study and that all information will be kept strictly confidential, except as required by law.

11.4 Data disclosure

No other entities will have access to this data.

11.5 Data safety and monitoring

The automated texting system staff is responsible for preventing unauthorized access to the trial participant tracking system database through its use of secure network firewall technologies. The study will utilize a client-server deployed Data Management System (DMS) rather than a 'Store and Forward' database configuration, obviating research site database security concerns. Confidential participant information will be entered into the database. If this information exists on paper CRFs, it will be filed under lock and key, with generation of a participant ID. Thereafter, confidential information will be made available to authorized users only as specifically needed. No one can gain access to an individual MySQL database table unless explicitly granted a user ID, password, and specific access. Even those with user names and passwords cannot gain access to the tables that contain the identifying participant information.

No results will be reported in a personally identifiable manner. All tracking system data will be password-protected with several levels of protection. The first will allow access to the operating system of the computer. The second will allow access to the basic menus of the integrated system; within certain menu options, such as database browsing, a third password will be required. Our prior research employing similar precautions has demonstrated that these techniques are very successful in assuring the protection of subjects.

Each investigator and staff member involved in the proposed study will sign and adhere to a Standard Operating Procedure for managing participant data through the automated texting platform and has participated in required IRB/HIPAA compliance training. We will also continue to make use of password protection programs for all computerized records. In no instances will identifying information be publicly disclosed. Prior to conducting any analyses, all identifiers (e.g., names, medical record numbers, health plan enrollee numbers, birth dates, etc.) will be removed. Results from this part of the investigation will be reported in aggregate. All participant identification will be removed from the data 3 years after the study has been completed.

11.6 Risk/benefit

11.6.1 Potential study risks

The anticipated potential risks for participants who are enrolled in this study are (1) harm resulting from a breach of confidentiality.

Harm resulting from a breach of confidentiality for all participants could include persons getting inappropriate access to interview/clinical data. Participants are at risk to be identified as someone who reports using opioids to manage pain.

Data collected in the study will be kept strictly confidential and will not be shared with anyone outside of the research team. The only exceptions to confidentiality, which will be clearly specified in the consent form, will be for information related to medical emergencies, current child abuse or neglect, or imminent risk of death or serious injury to the participant or others.

All project staff will be required to undergo training in confidentiality procedures (including HIPAA regulations) which review data handling procedures, as well as addressing questions about participants posed by individuals outside of the immediate project staff and by project staff members who do not need the information requested. Certification of successful completion of this training is kept in the appropriate project binder. Should any breaches of participant confidentiality occur during the course of the study, they will be reported to the relevant IRB, DSMB and NIDA officials.

11.6.2 Potential study benefits

Participation may increase their awareness of pain management, prescription opioid use and disposal. This is a low risk study and we believe the aforementioned risks are reasonable compared to the direct benefits.

11.6.3 Risk/benefit assessment

Anticipated risks of this study should be minimal and the risk/benefit ratio is very favorable.

Text Messaging Script

Consent: **Q1**

"Hi, this is Penn Medicine checking in about your recent procedure. Text 'YES' if we can ask a few questions about how you are managing your pain. Text 'STOP' anytime to opt-out. Click here: bit.ly/2HJCIg4 to learn more about this program.

Texting is not secure. Other people may be able to see information in text messages. By texting back 'YES' you are accepting this risk. Message & data rates may apply."

Q2:

We wanted to see how you are doing after your recent procedure. We may check in a few times.

[line break] How would you rate your pain over the past 24 hours? (from 0-10, 0 being no pain [please choose one number])."

LOGIC:

- If response ≠ 0-10 → REPLY "*I'm sorry. We don't understand. Please text back a number 0-10."*
- If no response in 3 hours → REPLY *"We missed your response. How would you rate your surgical pain over the past 24 hours? (from 0-10, 0 being no pain [please choose only one number]).*

Q3:

"We encourage you to contact your provider if your pain is too much to handle.

[line break] How have you been able to manage your pain since being home? From 0-10, 0 being not at all able

[please choose one number]."

LOGIC:

- If response =0-10 → Proceed to **Q4**
- If response ≠ 0-10 → REPLY "*I'm sorry. We don't understand. Please text back a number between 0-10."*
- If no response in 3 hours → REPLY *"We missed your response. How have you been able to manage your pain since being home? From 0-10, 0 being not at all able [please choose one number]."*

Q4:

"We just have a few more questions. [line break] Have you taken any of the following medications for your pain since your procedure (Acetaminophen, Aspirin, Ibuprofen, or Naproxen)? [Y for yes or N for no]. "

LOGIC:

- If response = Y, N, yes, or no → Proceed to **Q5**
- If response ≠ Y, N, yes or no → REPLY *"I'm sorry. We don't understand. Please text back Y or N."*

Q5:

"Thanks, this information helps guide our practice. It looks like you were also prescribed [opioid_RX] for your pain. [line break]Have you taken this medication? [Y for yes or N for no]."

LOGIC:

- If response = Y or yes → Proceed to **Q6**
- If response = N or no → Proceed to **Q10**
- If response ≠ Y, N, yes or no → REPLY *"I'm sorry. We don't understand. Please text back Y or N."*

Q6:

"Can you estimate how many pills of [opioid_RX] you have taken since your procedure? Please text back a number"

LOGIC:

- If response = Number→ Proceed to **Q7**
- If response ≠ Number→ REPLY *"I'm sorry. We don't understand. Please text back a number."*

Q7: *"Do you have any of these pills remaining? [Y/N]"*

LOGIC:

- If response = Y, yes → Proceed to **Q8**
- If response = N, no Proceed to **Q10**
- If response ≠ Y, N, yes or no → REPLY: *"I'm sorry. We don't understand. Please text back Y or N."*

Q8: *"Do you plan to continue taking [opioid_RX] to control your pain? [Y/N]"*

LOGIC:

- If response = Y or yes → REPLY: *"Thanks so much for your feedback! We'll check back with you in a few days. If you have any questions or concerns about your care or health please contact your surgical team. Have a nice day!"*

---This patient will be texted in 1 week---

- If response = N or no → **Move to Q9**
- If response ≠ Y, N, yes or no → REPLY: *"I'm sorry. We don't understand. Please text back Y or N."*

Q9: "Have you disposed of your unused %opioid% medication? [Y/N]

LOGIC:

- If response = Y or Yes → That's great to hear. It's important to dispose of your unused medication. Just one more question for you: [line break] *"How many days did it take for your pain to improve after your procedure? Please text back a number."*
- If response = N or No → It's important to dispose of your unused medication. Penn pharmacies will take them back. Find locations here: https://bit.ly/2VQPekA. [line break] Just one more question for you: *"How many days did it take for your pain to improve after your procedure? Please text back a number."*

Q10:

"Thank you so much! Just one more question for you. How many days did it take for your pain to improve after your procedure?"

LOGIC:

- If response = Number → REPLY "Thanks so much for your feedback! If you have any questions or concerns about your care or health, please contact your surgical team.
    - If response ≠ Number→ REPLY *"I'm sorry. We don't understand. Please text back a number."*
