# Supplementary material for: Effect of Mailing an At-home Disposal Kit on Unused Opioid Disposal After Surgery: A Randomized Clinical Trial
Source: JAMA Netw Open. 2022 May 6;5(5):e2210724. doi: 10.1001/jamanetworkopen.2022.10724 (PMC9077482; doi:10.1001/jamanetworkopen.2022.10724)
Supplement: Supplement 2. — eMethods. Study Population, Data Collection, Statistical Analysis, and Study Flowchart eReferences [file jamanetwopen-e2210724-s002.pdf]

Supplementary Online Content

Agarwal AK, Lee D, Ali Z, et al. Effect of mailing an at-home disposal kit on unused opioid disposal after surgery: a randomized clinical trial. *JAMA Netw Open*. 2022;5(5):e2210724. doi:10.1001/jamanetworkopen.2022.10724

eMethods. Study Population, Data Collection, Statistical Analysis, and Study Flowchart

eReferences

This supplementary material has been provided by the authors to give readers additional information about their work.

© 2022 Agarwal AK et al. *JAMA Network Open*.

eMethods. Study Population, Data Collection, Statistical Analysis, and Study Flowchart

Study Population

Through an on-going institutional quality improvement effort, all patients undergoing orthopedic and urologic surgical procedures at the Hospital of the University of Pennsylvania and Penn Presbyterian Medical Center are invited to participate in a post-operative program using text messaging. The details of this program have been previously published on engaging patients using text messaging and capturing patient-reported outcomes data including self-reported pain intensity, use of opioid prescription tablets, and disposal.[1,2] Patients are considered eligible if they are 18 years or older, are prescribed an opioid for postoperative control of acute pain, and had a verified mobile telephone number listed in the electronic health record. This program and previous studies, including this study have been approved by the institutional review board of the University of Pennsylvania.

Data Collection

Eligible patients are identified from automated daily operative reports of patients undergoing surgical procedures and organized by department. The telephone number of each eligible patient is automatically entered into an automated text messaging platform, which is programmed to prospectively collect data from patients on patient-reported outcomes (e.g. pain intensity, ability to manage pain), opioid use, and disposal in the 28 days following the procedure. The initial text message obtains electronic consent to receive text messages and collect self-reported data. This process of electronic written consent via text messaging has been approved by the University of Pennsylvania Health System's privacy and safety committee and the University of Pennsylvania institutional review board. Patients have the ability to opt out at any time by replying "STOP."

After consent, participants are asked to self-report pain intensity on a scale of 0 to 10, with 10 being the highest level of pain; their ability to manage pain on a scale of 0 to 10, with 10 representing very able to control pain; their prescription opioid use in number of tablets and; if they disposed of any unused tablets. Patients are asked whether they plan to take opioids to manage pain in the upcoming days. Patients reporting current or planned future opioid use are queried on subsequent days 7, 14, 21, and 28. When a patient reported no use or no further use, text messaging ended. Prescribers are not directed to change their standard of care when discussing pain management, use of analgesics, or prescribing practice. In addition, text message questions are not framed to provide clinical guidance on managing pain but rather as means to collect patient-reported scores of pain intensity, ability to manage pain, and use of medication. Patient information is deidentified and stored securely. Demographic information, comorbidities, mental health history, and type of surgical procedure were obtained from the electronic health record. Opioid naïve patients are defined as no opioid prescriptions within the electronic health record over the past year, similar to other published reports.[2]

Statistical Analysis

The primary outcome of interest was self-reported opioid. R, version 3.60 (R Project for Statistical Computing) was used for statistical analysis. A 2-sided α of .025 was considered statistically significant. Descriptive statistics were used to summarize patient demographic characteristics. Comparisons were done with the Fisher exact and χ^2 tests for categorical variables and Kruskal-Wallis test for continuous variables. Patients who did not respond, or missing data, were classified as not reporting opioid disposal.

Patients self-reported disposal via text messaging survey. Baseline self-reported disposal rates were 25-30%. We estimated 75 patients per group, assuming a two-sided alpha of 0.025, a beta of 80%, and a 50% relative increase in self-reported disposal. The primary outcome of interest was self-reported disposal. All randomized participants were included in an intention-to-treat analysis.

© 2022 Agarwal AK et al. *JAMA Network Open*.

Study Flowchart

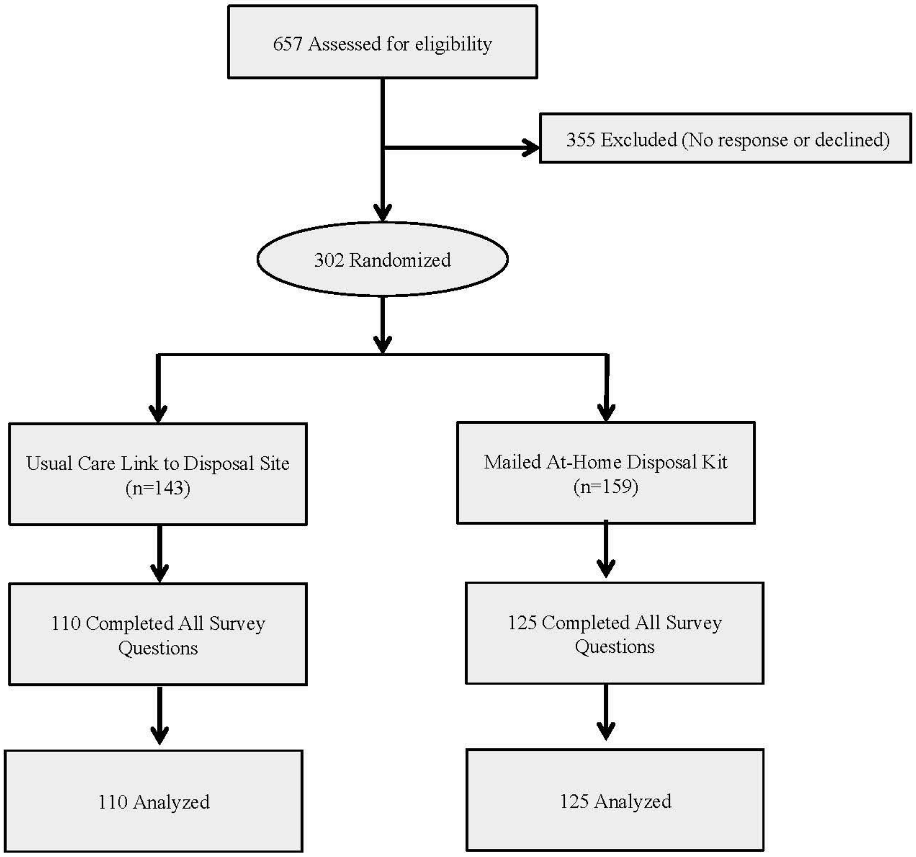

© 2022 Agarwal AK et al. *JAMA Network Open*.

eReferences

1. Brummett CM, Steiger R, Englesbe M, et al. Effect of an activated charcoal bag on disposal of unused opioids after an outpatient surgical procedure: a randomized clinical trial. JAMA Surg. 2019;154(6):558-561. Medline:30916733 doi:10.1001/jamasurg.2019.0155</jrn>

2. Agarwal AK, Ali ZS, Sennett B, et al. An automated text messaging program to inform postoperative opioid prescribing. NEJM Catalyst Innovations in Care Deliv. Published online February 17, 2021. doi:10.1056/CAT.20.0440

© 2022 Agarwal AK et al. *JAMA Network Open*.
