# Supplementary material for: Effect of Mailing an At-home Disposal Kit on Unused Opioid Disposal After Surgery: A Randomized Clinical Trial
Source: JAMA Netw Open. 2022 May 6;5(5):e2210724. doi: 10.1001/jamanetworkopen.2022.10724 (PMC9077482; doi:10.1001/jamanetworkopen.2022.10724)
Supplement: Supplement 3. — Data Sharing Statement [file jamanetwopen-e2210724-s003.pdf]

Data Sharing Statement

Agarwal. Effect of Mailing an At-home Disposal Kit on Unused Opioid Disposal After Surgery. *JAMA Netw Open*. Published May 06, 2022. doi:10.1001/jamanetworkopen.2022.10724

Data
Data available: Yes
Data types: Deidentified participant data
How to access data: Available upon request, anish.agarwal@pennmedicine.upenn.edu
When available: With publication

Supporting Documents
Document types: None

Additional Information
Who can access the data: Researchers whose proposed use of the data has been approved)
Types of analyses: For any purpose
Mechanisms of data availability: With investigator support, after approval of a proposal and with a signed data access agreement.
Any additional restrictions: none.
